# The Migration Mechanism of BTEX in Single- and Double-Lithology Soil Columns under Groundwater Table Fluctuation

**DOI:** 10.3390/toxics11070630

**Published:** 2023-07-20

**Authors:** Jingwei Zheng, Yang Yang, Juan Li, Hao Zhang, Yan Ma

**Affiliations:** 1 School of Chemical and Environmental Engineering, China University of Mining and Technology-Beijing, Beijing 100083, China; zjwljwater@foxmail.com; 2 Technical Centre for Soil, Agriculture and Rural Ecology and Environment, Ministry of Ecology and Environment, Beijing 100012, China; zhanghao@tcare-mee.cn

**Keywords:** groundwater table fluctuation, benzene and toluene, migration mechanism, single- and double-lithology soil columns

## Abstract

The migration of light non-aqueous phase liquids (LNAPLs) trapped in porous media is a complex phenomenon. Groundwater table fluctuation can not only affect contaminant migration but also redox conditions, bacterial communities, and contaminant degradation. Understanding LNAPLs’ (e.g., benzene, toluene, ethylbenzene, and xylene (BTEX)) behavior within porous media is critical for the high efficiency of most in situ remediation systems. A laboratory study of single- and double-lithology soil column investigation of the groundwater table fluctuation effect on BTEX transport, using benzene and toluene as typical compounds, in a typical representative model of aquifers subjected to water table fluctuation was undertaken in this study. The results show that benzene and toluene migration in single-lithology soil columns packed with sand was mainly affected by flushing due to the hydraulic force induced by water table fluctuations and that the double-lithology soil column packed with sand and silt was significantly affected by retention due to the higher adsorption induced by 10 cm of silt. The dissolution mainly correlated with the BTEX migration in saturated zones, and the contaminant concentration increased when the water table fell and decreased when the water table rose. For a contaminated site with a single-lithology structure consisting of sand, more attention should be paid to organic contaminant removal within the groundwater, and a double-lithology structure containing silt is more suited to the removal of organic contaminants from the silt layer. The difference in biodegradation kinetics between the groundwater table fluctuation (GTF) zone and the saturated zone should be better understood for the remediation of BTEX compounds.

## 1. Introduction

Subsurface contamination by non-aqueous phase liquids (NAPLs) is a widespread problem. NAPLs cause concern because of their persistence in the subsurface and their ability to contaminate large volumes of soil and groundwater. NAPLs that are lighter than water, such as gasoline and BTEX, are referred to as light non-aqueous phase liquids (LNAPLs) [1]. LNAPLs present extensive contamination risks due to their ability to partition into both air and water phases, creating pathways for exposure to human health and the environment [2]. Volatile petroleum hydrocarbons (PHCs) released in the form of vapor from LNAPLs, known as petroleum vapor intrusion (PVI), represent the most likely pathway for human health risks [3]. In recent years, significant efforts have been made to optimize the development of effective methods for the remediation of contaminants in soils and groundwater, such as air sparging and bioventing [4]. However, these techniques can be expensive and, in some cases, ineffective [4]. Significant efforts have been made in recent years to develop effective methods to remove pollutants from contaminated soil and groundwater. Not only have chemical and biological interactions been researched [5] but studies have also focused on the diffusion and migration of pollutants in groundwater [6,7]. Pollutant behavior within porous media is critical for the high efficiency of most in situ remediation systems [8].

The descriptions of water flow and phase distribution are the basis for understanding the transport of dissolved organic pollutants from the soil surface to groundwater. When a small amount of an LNAPL is released into the subsurface, it migrates through the unsaturated zone until it distributes itself as a residual saturation. If the release is large enough, it reaches the capillary fringe, where it spreads laterally above the saturated zone [9]. The outcome for organic chemicals not only depends on their interactions with the solid phase but also on the water flow velocity in the soil [10,11]. Water flow largely depends on the amounts and intensities of precipitation and evapotranspiration, as well as the movement of the free water table in the subsoil or aquifer. Drying and wetting processes driven by water potential differences may occur simultaneously at different depths under field conditions [12,13]. The groundwater table fluctuation (GTF) zone, where the groundwater table rises and falls due to the removal or recharge of groundwater, tidal patterns, or industrial activities has a significant effect on contaminant migration. However, little is known about the impact of GTF zones on contaminants [14,15]. The GTF zone causes drying and wetting processes, which, linked to the redox of hydrochemistry [16], can lead to the dissolution and subsequently more complicated transport of petroleum (e.g., benzene and toluene) in contaminated subsurface environments. The rise and fall of the groundwater table may promote contaminant migration through the groundwater level, where the contaminants pass through the water boundary and contaminate the lower groundwater regions, resulting in pollution plume dispersion [17,18].

With repeated rises and falls due to GTF, LNAPLs that float on the surface layer are particularly susceptible to adsorption into the porous region surrounding the water table, known as the smear zone [19,20]. Previous studies have shown that water level fluctuation has a significant influence on the migration of organic pollutants throughout the water table [21], suggesting that the dissolution and biodegradation of LNAPL components is likely to increase [22]. Due to the potentially widespread and dispersed nature of petroleum hydrocarbon plumes, the remediation process can be lengthy, requiring cost-effective and practical methods to ensure the long-term control of contaminated groundwater [23]. Compared to other remediation techniques, natural attenuation (NA) has been recognized as one of the remediation options that can meet the aforementioned requirements [24]. NA is a passive remediation measure that relies on natural mechanisms to degrade and remove contaminants from the soil and groundwater [25]. Due to its cost-effectiveness and minimal environmental impact, NA is considered a potential approach for the remediation of sites contaminated with petroleum hydrocarbons [26]. Within intrinsic biotic and abiotic mechanisms, intrinsic bioremediation (including natural aerobic and anaerobic biodegradation processes) plays key roles in plume control and pollutant removal [26]. The biodegradation of petroleum hydrocarbons occurs naturally with indigenous microbes and is an inexpensive and sustainable option [27]. During biodegradation, petroleum hydrocarbons break down into less hazardous substances. The whole mass loss of LNAPLs due to partitioning and physical and biological degradation is termed natural source zone depletion (NSZD) [28]. Many studies have investigated the microbial communities and physical conditions that may lead to the natural source zone depletion (NSZD) of petroleum hydrocarbons [29]. These studies have considered the roles of different redox conditions in biodegradation [30] and how groundwater fluctuations can cause changes in the redox environment, thereby promoting microbial degradation [31]. Therefore, it is essential to better understand the behavior and transformation mechanisms of organic pollutants in water table fluctuation zones in order to improve the effectiveness of remediation methods for groundwater pollutants.

GTF can not only affect contaminant migration but also redox conditions, bacterial communities, and contaminant degradation. Water table cycle fluctuations can accelerate the attenuation of pollutants in groundwater [32]; for example, the removal rate of NH_4_-N has been found to be increased by higher rates of water level fluctuation [1], and diesel oil has also been found to be increased by GTF [33]. The redox conditions for a fluctuating water table were not in equilibrium but were subjected to spatial and temporal variations. The microbial processes in the column that was well-defined in terms of water flow, gas flow, and initial mineral phase resulted in heterogeneity that had consequences for organic pollutants [34].

The toxic aromatic hydrocarbons benzene, toluene, ethylbenzene, and m-xylene (BTEX) are common petroleum pollutants in the subsurface environment [35]. Due to the low density and solubility of benzene and toluene, they behave as LNAPLs and are absorbed into the surface layer of the water table. The migration of benzene and toluene to the subsurface environment, e.g., advection, dispersion, adsorption, and degradation [36], is mainly affected by the flow patterns and porous media characteristics. Field investigation studies have shown that cyclical fluctuations have a significant influence on the migration and distribution of BTEX in groundwater [37,38]; however, the migration mechanisms of BTEX associated with the hydraulic force and different soil structures of GTF have rarely been studied.

Therefore, this study explored the BTEX migration mechanism in a laboratory soil column GTF system. Benzene and toluene was chosen as BTEX compound. Two typical porous media were used in the experiment, consisting of single- and double-lithology soil columns. This simulated groundwater table system allows investigation of the effects of hydraulic force and allows investigation of different porous media characteristics controls on a representative subunit of a GTF system to further understand benzene and toluene migration from petroleum-contaminated shallow groundwater and the effects of geological media, as well as to identify benzene and toluene abundance zone and assist strategies for the remediation of benzene and toluene compounds.

## 2. Methodology

### 2.1. Conceptual Model for Pollutants in Water Table Fluctuation Zone

**Figure 1:**
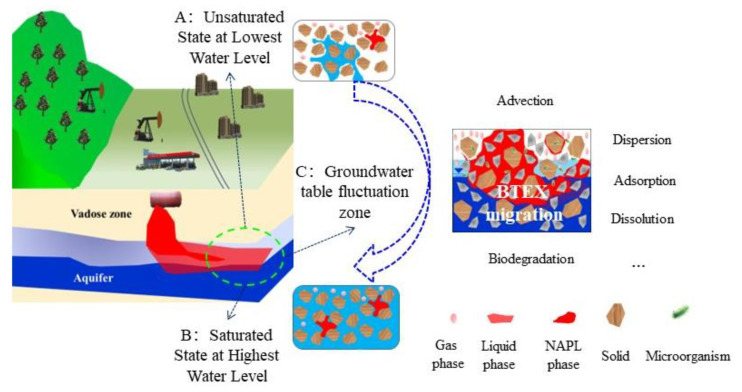
Conceptual model of petroleum contamination in groundwater fluctuation zones. Saturated state at highest water level (**A**); unsaturated state at lowest water level (**B**); groundwater table fluctuation zone (**C**). Red represents the pollutant and blue represents groundwater.

Generally, once organic pollutants are released into the subsurface, most migrate downwards through the unsaturated vadose zone [21,22] to the capillary fringe and groundwater table [22]. Since shallow subsurface environments can be saturated or unsaturated, the migration and transformation of pollutants via different soil layers are affected by a large number of different environmental factors, particularly in GTF zones with frequently alternating cycles of saturation and unsaturation. The transport and fate of benzene and toluene in the subsurface were subject to four main fundamental mechanisms as follows: the infiltration and migration of benzene and toluene in the subsurface under the influence of gravitational and capillary forces, the dissolution and consequent advection–dispersion in the downward-flowing water phase, the transport of benzene and toluene vapor as the soil pore gas, and the influence of chemical and biological functions on benzene and toluene. As shown in Figure 1, when the groundwater table rises, the porous media becomes saturated and contaminants can be dispersed or caused to migrate with the water flow (Figure 1A); when the groundwater table falls, the porous media becomes unsaturated and the residual contaminants can be suspended in a three-phase gas–liquid–solid state (Figure 1B).

### 2.2. Experimental Setup

**Figure 2:**
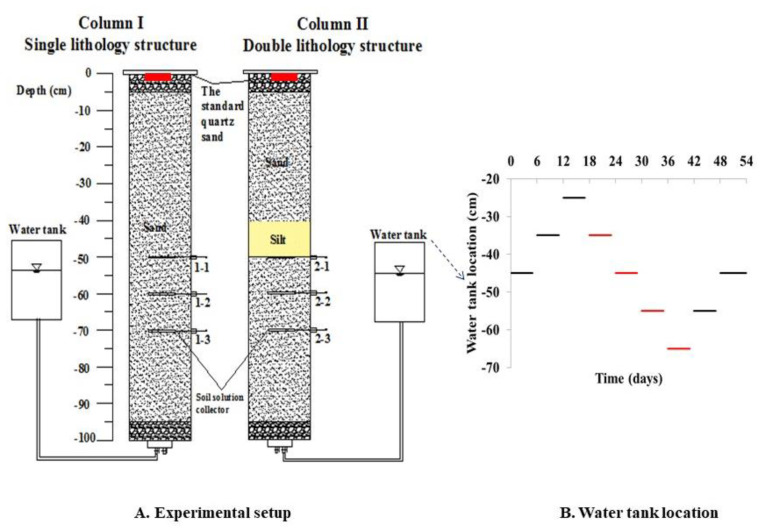
In the schematic of the experimental apparatus (**A**), red is the leakage point of pollutants, and in the water table location (**B**), red represents the stage of water level decline.

A schematic diagram of the experimental setup is shown in Figure 2, and it is used to simulate the GTF zone. The migration mechanisms of benzene and toluene were assessed under varying hydraulic conditions, including single- and double-lithology soil and groundwater table fluctuation conditions. The columns were made of organic glass tubes (height of 100 cm, from top 0 cm to bottom −100 cm; diameter of 10 cm). Pretreated sand and silt were uniformly packed into the columns. Column I was a single-lithology structure packed with sand only. Column II was a double-lithology structure packed with sand and silt, and the silt was located in the middle of the column. A total of 600 mL of pure benzene (300 mL) and toluene (300 mL) liquid was evenly loaded into each column, and the top of the column was sealed immediately to prevent loss of benzene and toluene by evaporation [39].

To simulate the hydraulic dynamic conditions experienced in the GTF zones, the water tank location was adjusted to represent the change in the groundwater table. The amplitude of the fluctuation was 20 cm, and the whole duration of the experiment was 54 days. At the beginning of the experiment, the groundwater table was −45 cm from 0 to 6 days, −35 cm from 7 to 12 days, −25 cm from 13 to 18 days, −35 cm from 19 days to 24 days, −45 cm from 25 days to 30 days, −55 cm from 31 days to 36 days, −65 cm from 37 days to 42 days, −55 cm from 43 days to 48 days, and −45 cm from 49 days to 54 days. Each water sample was collected at the end of each groundwater level using a soil solution collector (Rhizon, Holland). The locations of the collected water samples were at −50 cm (Column I 1–1 and Column II 2–1), −60 cm (Column I 1–2 and Column II 2–2), and −70 cm (Column I 1–3 and Column II 2–3). The number of water samples collected by column I and column II was 9 and 9, respectively. Four soil samples from each column were collected to assess the groundwater bacterial community characteristics, and the locations of the soil samples were at −30 cm, −40 cm, −50 cm, and −60 cm, respectively.

### 2.3. Simulation Materials

Two types of porous media were prepared. Column I was packed with sand only, and column II was packed with sand and silt. Table 1 summarizes the basic characteristics of the porous media. The simulated groundwater was collected from a shallow aquifer without benzene and toluene contamination (below the detection limit). And the physical and chemical characteristics of benzene and toluene are listed in Table 2.

### 2.4. Analytical Methods

A total of 18 water samples were collected from the columns during the whole experiment process. Water samples from the columns were analyzed for benzene and toluene concentrations. Benzene and toluene concentrations were analyzed using gas chromatography–mass spectrometry (GC-MS 2010QP-Ultra, SHIMADZU, Japan). The temperature of the chromatographic column was 40 ℃. Groundwater bacteria were analyzed using high-throughput sequencing, and the genomic DNA of all tiny organisms in the environmental sample was tested using a shotgun [42,43].

## 3. Results

### 3.1. Benzene and Toluene Change with Depth

**Figure 3:**
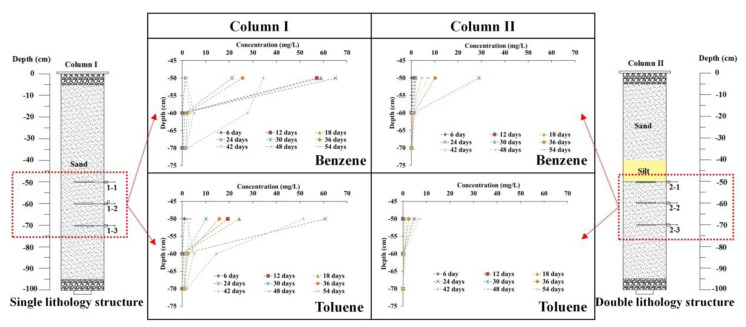
The concentration of benzene and toluene changes with soil column depth.

Benzene and toluene concentration variation along with the depth of the column are compared in Figure 3 for column I and column II. The change range in benzene concentration in column I was 1.26 to 65.16 mg/L, 0.0064 to 5.73 mg/L, and 0.0011 to 1.97 mg/L at 1–1, 1–2, and 1–3, respectively; in column II, it was was 0.25 to 28.98 mg/L, 0.00027 to 1.52 mg/L, and 0.00044 to 0.42 mg/L at 2–1, 2–2, and 2–3, respectively. The change range in toluene concentration in column I was 0.99 to 60.68 mg/L, 0.0025 to 14.44 mg/L, and 0.0007 to 1.84 mg/L at 1–1, 1–2, and 1–3, respectively; in column II, it was was 0.0074 to 4.99 mg/L, 0.00025 to 0.88 mg/L, and 0.00038 to 0.24 mg/L at 2–1, 2–2, and 2–3, respectively. The amplitude of benzene and toluene concentration variation for column I was almost twice or more than that shown in column II with the addition of 10 cm silt, and the concentration at the same depth for the column was higher than that in column II, showing the additional contaminant retention provided by silt in the porous media in comparison to sand media alone. Therefore, the concentration amplitude for sand only was also faster than that for the porous media containing silt, highlighting the negative impact on BTEX migration in column II due to the inclusion of silt in the porous media. When comparing the BTEX migration mechanism between porous media types, the contaminant concentration amplitude was higher in the media composed of sand only than in the porous media containing silt.

(1) $$logK_{OC}=0.623logK_{OW}+0.873$$

*Koc* represents the soil/sediment sorption coefficient. *K_OW_* is the water/octanol partition coefficient [44].

(2) $$C_{soil}=C_{water}\times K_{oc}$$

*C_soil_* represents the solute concentration in soil, *C_water_* represents the solute concentration in water, and *K_oc_* represents the organic carbon–water partition coefficient in soil/sediment [45].

**Figure 4:**
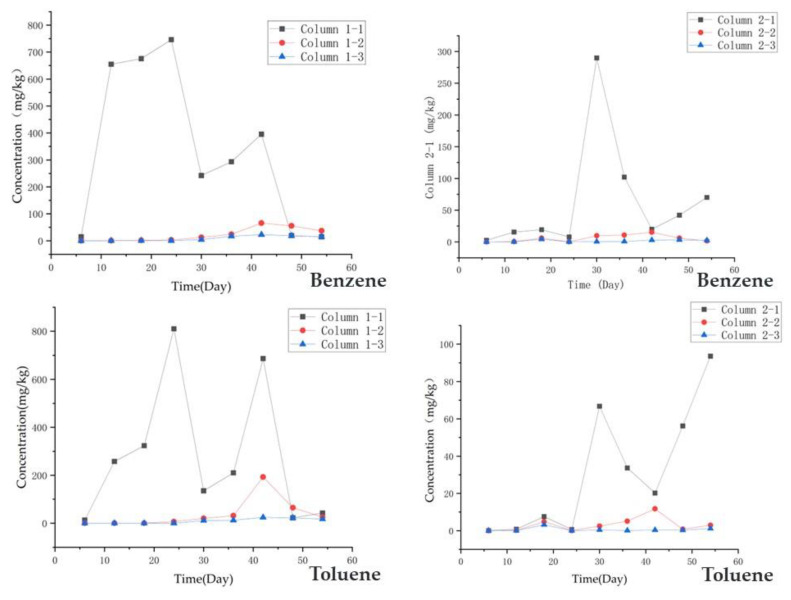
The concentration changes of benzene and toluene in the soil.

Using Equation (1), the *Koc* value can be calculated. Using Equation (2), the concentrations of benzene and toluene in the soil can be calculated. Figure 4 shows the predicted concentrations of benzene and toluene in the soil. The highest predicted concentration of benzene in the soil was 746 mg/kg for column 1 and 331 mg/kg for column 2. The highest predicted concentration of toluene was 686 mg/kg for column 1 and 93 mg/kg for column 2. As the water level fluctuated, the pollutant concentrations gradually decreased. This may be associated with microbial activity [46].

### 3.2. Benzene and Toluene Changes with GTF and Bacterial Community Structure

**Figure 5:**
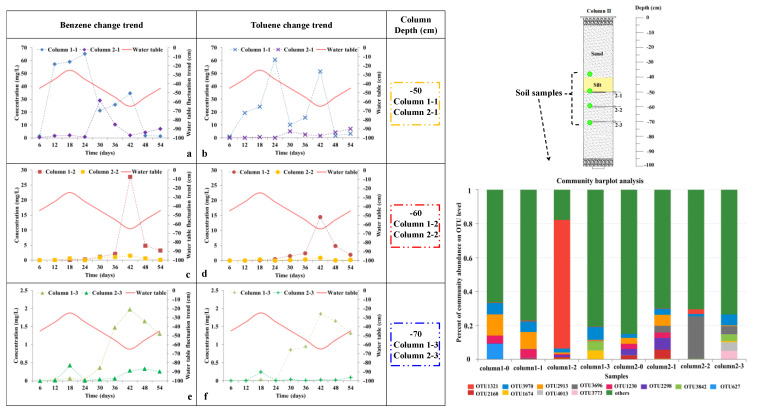
The benzene and toluene concentration changes under groundwater table fluctuation, and the bacterial community with depth after the experiment.

Water samples from different depths (−50 cm, −60 cm, −70 cm) in the two columns were analyzed for BTEX change trends under water table fluctuations, as shown in Figure 5 (left). In general, benzene and toluene concentrations at the same depth showed the same change trends in columns I and II with water table fluctuation, showing a higher concentration amplitude in column I. The benzene and toluene concentrations always changed with the water table fluctuation at −50 cm in the whole period, showing a lower concentration amplitude in the initial period followed by a higher amplitude at −60 and −70 cm after several water table up-down cycles. Notable differences were observed between column I and column II. First, the BTEX concentration showed a higher trend in column I than that in column II, especially at −50 cm, despite the same concentration and volume of benzene and toluene being applied to each column. The highest benzene and toluene concentrations were 65.16 mg/L and 60.68 mg/L in column I, 28.98 mg/L and 4.99 mg/L in column II, and almost all the concentrations of the samples in column II were lower than that in column I. Thereafter, there were different changes in benzene and toluene concentrations between column I and column II, especially in the whole period at −50 cm, and in the last period at −60 cm and −70 cm.

The benzene concentration change trends at −50 cm, −60 cm, and −70 cm are shown in Figure 5a,c,e, respectively. As shown in Figure 5a, when the groundwater table rose by 20 cm from 6 days to 18 days, the benzene concentration noticeably increased in column 1–1 and slightly in column 2–1, and the increased amplitude was 57.73 mg/L and 1.67 mg/L. When the groundwater table declined by 40 cm from 18 days to 42 days, the benzene concentration initially decreased, followed by an increase in column 1–1; however, it initially increased followed by a decrease in column 2–1. When the groundwater table rose by 20 cm from 42 days to 54 days, the benzene concentration decreased in column 1–1 and increased in column 2–1, showing the opposite trend. As shown in Figure 5c,e, the benzene concentration was stabilized from 6 days to 24 days. Thereafter, the benzene concentration increased as the groundwater table declined from 24 days to 42 days and decreased as the groundwater table rose from 42 days to 56 days.

The toluene concentration change trends at −50 cm, −60 cm, and −70 cm were shown in Figure 4b,e,f, respectively. The toluene concentration change trend was similar to that of benzene at −50 cm, −60 cm, and −70 cm. The main reason for the benzene and toluene concentration change trends may be the 10 cm silt in column II, which changed the soil column structure, including hydrodynamic force, permeability coefficient, adsorption, and biodegradation conditions. And the sample points were at different depths; columns 1–1, 1–2, 2–1, and 2–2 were in the groundwater table fluctuation zone with “unsaturation and saturation cycles”, and columns 1–3 and 2–3 were in the saturation zone. Therefore, different points underwent different water conservation conditions.

Additionally, soil samples were also collected from different depths in two columns for bacterial community analysis after the experiment, as shown in Figure 5 (right). The compositions and abundance of the bacterial communities at the OTU level are shown as −40 cm (columns 1–0 and 2–0), −50 cm (columns 1–1 and 2–1), −60 cm (columns 1–2 and 2–2), and −70 cm (columns 1–3 and 2–3). Three samples were collected at each depth for the repeatability test. The classified sequences at each depth were affiliated with 13 bacterial OTUs (>5 %). For all 12 OTUs except others (green color in Figure 5), OTU3970 belonged to Actinobacteria, OTU2168 belonged to Cyanobacteria, OTU3696 was unclassified, and the others in 12 OTUs belonged to Proteobacteria at the phyla level. For column I and column II, OTU3970 existed along with depth, and at each depth, the approximate relative abundances were larger than 1%. OTU2913 and OTU1230 also existed in the two columns; however, the relative abundances were obviously higher at the top of the GTF zone (−40 and −50 cm) than that at the bottom of the GTF zone (−60 and −70 cm). Additionally, OTU3824 relative abundances were obviously higher at −70 cm.

For column 1–0 and column 2–0, the differences were OTU627 (9.0%) in column 1–0, and OTU2298 (3.5%) and OTU2168 (2.6%) in column 2–0. For column 1–1 and column 2–1, the differences were OTU3696 (3.8%) in column 1-1, and OTU2298 (6.9%) and OTU2168 (5.3%) in column 2–1. For column 1–2 and column 2–2, the differences were OTU1321 (76%) in column 1–2, and OTU1321 (2.9%) and OTU3696 (25%) in column 2-2. For column 1–3 and column 2–3, the differences were OTU1674 (5.3%) in column 1–3, and OTU3696 (4.3%), OTU4013 (5.3%), and OTU3773 (5%) in column 2–3. Significant variations were detected between column I and column II.

## 4. Discussion

### 4.1. Single- and Double-Lithology Structure Effect on BTEX Migration Mechanisms

Column I was a single-lithology structure with sand only, and column II was a double-lithology structure with sand and 10 cm silt. The benzene and toluene concentrations in column 2–1 were significantly lower than those in column 1–1 during the whole GTF period, showing a higher level of benzene and toluene retention within the porous media of silt. Therefore, a contaminated site with a double-lithology structure containing silt was more contaminated with BTEX, and a contaminated site with a single-lithology structure consisting of sand was more suited for the removal of organic contaminants from within the groundwater.

The benzene and toluene retention within the silt has a significant effect on BTEX migration, mainly because of BTEX adsorption and dissolution from the porous media. Adsorption and dissolution are influenced by hydro-chemical and geological characteristics [39,47,48]. Parameters such as pH, initial porosity, specific surface area, organic matter content, and media size were subjected to factor analysis. The basic physical and chemical properties of porous media are shown in Figure 3. Compared to sand, silt has a smaller media size and initial porosity, which indicates that silt has a higher specific surface area. Additionally, silt has higher organic matter content. Notably, the differences in these parameters show the different effects of sand and silt on benzene and toluene migration. When assessing the correlation of the parameters of media size with benzene and toluene adsorption, there was a negative correlation. Smaller media sizes will have a higher specific surface area, which leads to easier adsorption of benzene and toluene on silt than on sand. Therefore, the media size and specific surface area were significantly correlated with the benzene and toluene adsorption ratio but showed an inverse correlation with benzene and toluene dissolution. However, the initial porosity of the porous media was positively correlated with benzene and toluene dissolution. The initial porosity of porous media was higher, and benzene and toluene were more easily desorbed from the porous media. Therefore, due to the silt in column II, benzene and toluene concentrations were significantly lower than those in column 1–1 during the whole GTF period. This confirms that the dissolution and transport actions of the contaminants within the water could be reduced with higher levels of adsorption of contaminants into porous media [49].

### 4.2. Water Table Fluctuation Effect on BTEX Migration Mechanisms

**Figure 6:**
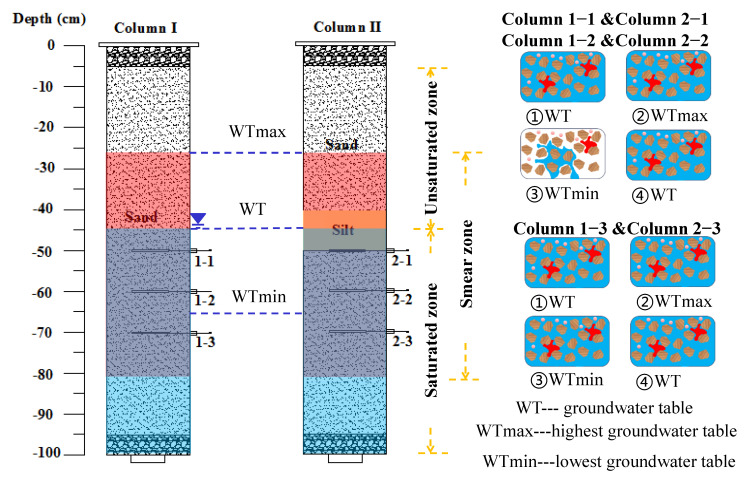
Schematic water table fluctuation with respect to soil column and porous media state. The blue arrow indicates the groundwater level, and the yellow arrow indicates the saturated and unsaturated zone of the groundwater.

The schematic water table fluctuation in the soil column and the porous media state are shown in Figure 6. The migration mechanism and distribution characteristics of BTEX in the soil column are discussed via image analysis. During the water table fluctuations, the contaminated porous media experienced periods of saturation and unsaturation, so contaminants such as benzene and toluene were in a continuous state of transformation between saturated and unsaturated environments. In theory, in this scenario, benzene and toluene exist in three different states: free, dissolved, and residual [8]. According to benzene and toluene solubility in 1800 mg/L and 347~707 mg/L of water at 25 ℃ in Table 2, the benzene and toluene concentrations of all the water samples did not exceed the criterion. The highest concentrations of benzene and toluene measured in column I were 65.16 mg/L and 60.68 mg/L, respectively, whereas in column II, they were 28.98 mg/L and 4.99 mg/L, respectively. These results are related to the adsorption of benzene and toluene by the soil and soil organic matter. Research by Jing Sun et al. showed that soil with an average particle size of 0.08 mm has a maximum adsorption capacity of 376.39 mg/kg for benzene [50]. The sorption behavior of soils also depends on the chemical conformation and physical structural properties of the soil organic matter (SOM) fraction [51]. Among the various factors influencing the sorption behavior, SOM is considered a key factor controlling the adsorption process as the distribution phase for NAPL uptake [52]. For example, Lion et al. suggested that the organic carbon (f_oc_) was a dominant factor controlling the sorption capacity of American soils even if f_oc_ < 0.1% [53]. Therefore, the dissolved benzene and toluene would migrate with the water flow, where the dissolution of contaminants has a significant impact on pollutant migration. In addition, benzene and toluene are light non-aqueous phase liquids; if the volume of contaminants is enough, they would form a lens above the groundwater table, and some contaminants would migrate with the water flow in free form. Therefore, groundwater table fluctuation would generate a hydrodynamic force, and free and dissolved toluene would be transported with the water flow. The residual state would adsorb onto the porous media and could not be transported with the water flow. However, the saturated and unsaturated environments of the porous media would cause benzene and toluene to migrate among free, dissolved, and residual states, leading to benzene and toluene concentrations in water fluctuation.

Soil water collectors at different depths experienced diverse saturation and unsaturation conditions. As shown in Figure 6, columns 1–1, 2–1, 1–2, and 2–2 experienced saturation from the water table (WT) to the highest water table (WTmax), unsaturation from WTmax to the lowest water table (WTmin), and then to saturation from WTmin to WT. Columns 1–3 and 2–3 always experienced saturation from WT to WTmax, and from WTmin to WT. Therefore, the benzene and toluene concentration fluctuations were different, as shown in Figure 5 (left).

For column I, the BTEX formed a lens above the water table and existed as free contaminants, and benzene and toluene dissolved in water. On day 6, the concentration was very low at −40, −50, and −60 cm. As the water table rose from WT to WTmax, the contaminant concentration in column 1–1 increased. Because the residual contaminants in the unsaturated zone were only occasionally in contact with water, these contaminants were less depleted in water, so more contaminants were transferred from the free and residual state to dissolve into the water and, consequently, the contaminant concentration increased as the water table increased. However, the contaminant concentrations in columns 1–2 and 1–3 remained stabilized because the water table increased, and free and residual state contaminants could not contact these depths. As the water table fell from WTmax to WTmin, the contaminant concentrations in column 1–1 decreased initially and thereafter increased. Because water initially promoted the dilution of dissolved state contaminants, as the water table fell, free and dissolved contaminants were transported with the water flow, and contaminant concentrations increased. Also, in columns 1–2 and 1–3, as the water table fell, the contaminant concentrations increased at the end of the water table fall period. This is because more dissolved state contaminants were transported to the depth with the water flow. As the water table rose from WTmin to WTmax again, the contaminant concentrations in columns 1–1, 1–2, and 1–3 decreased. As the water table rose, it promoted the dilution of the dissolved state contaminants.

For column II, the contaminant concentrations in columns 2–2 and 2–3 presented similar fluctuation trends to columns 1–2 and 1–3, and the concentration was smaller than that in column I. Although the contaminant concentration in column 2–1 was smaller than that in column 1–1, it presented different fluctuation trends, especially in the water table fall period from WTmax to WTmin. The 10 cm silt in column II, which had a higher retention of contaminants, led to this difference. At the beginning of the water table fall period, some dissolved and residual state contaminants were transported with the water flow to column 2–1, and the contaminant concentration increased. However, later in the water table fall period, more free-state contaminants were adsorbed onto the porous media, and fewer contaminants were transported with the water flow; as the water promoted the dilution of dissolved state contaminants, the contaminant concentration decreased.

Therefore, the benzene and toluene migration was mainly affected by flushing due to the hydraulic force induced by the water fluctuations, causing dissolution and adsorption onto porous media [7]. Because silt had a higher retention of benzene and toluene, which affected the benzene and toluene migration mechanism, more free and dissolved benzene and toluene adsorbed onto the porous media and formed residual benzene and toluene, and the effect of GTF on benzene and toluene migration in column I was higher than that in column II for the 10 cm silt. For a contaminated site with a single-lithology structure consisting of sand, GTF led to the downward migration of benzene and toluene, and the contaminant concentration in the saturated zone increased, which is why more attention should be paid to organic contaminant removal within the groundwater. A contaminated site with a double-lithology structure containing silt was more suited to the removal of organic contaminants from the silt layer.

### 4.3. Bacterial Community Structure Effect on BTEX Migration Mechanisms

As shown in Figure 5, the bacterial OTU numbers and abundance at each depth were significantly different. However, most of them belonged to Proteobacteria, which was also detected in many BTEX-contaminated sites. Proteobacteria are one of the main BTEX-degrading bacteria [54]. Biodegradation is an important process that can affect the dissolved state of the BTEX concentration, which affects BTEX migration. Biodegradation kinetic changes in response to water table fluctuations were demonstrated in several studies, e.g., Rainwater et al. (1993), Sinke et al. (1998), Dobson et al. (2007), and Rezanezhad et al. (2014) [33,34,55,56]. These studies suggest that biodegradation enhancement during episodes of a rising water table is attributed to aquifer oxygenation [38], and biodegradation mainly occurs under aerobic conditions. But some studies show that under anaerobic conditions, BTEX can also be degraded [57]. Because the GTF and soil lithology structures affect the aerobic and anaerobic conditions, the bacterial community presented obvious differences.

Therefore, it is speculated that oxygen is enough in the unsaturated zone and that aerobic biodegradation and hydrodynamic forces affect BTEX migration. In the GTF zone, the aerobic and anaerobic conditions always change, and biodegradation kinetics may be lower than the hydrodynamic force, which would mainly affect BTEX migration. In the saturated zone, anaerobic biodegradation and hydrodynamic forces affect BTEX migration. Additionally, it is speculated that under some conditions during the GTF period, biodegradation kinetics become higher than the mass transfer coefficient caused by the hydrodynamic force, which results in a strong reduction in the BTEX concentration.

Mass transfer continually removes the BTEX mass from LNAPL entrapped in the pores, with the aqueous BTEX mineralized by native bacterial metabolism. The quantification of BTEX mass removal is a key issue in natural source zone depletion (NSZD) assessment (ITRC, 2009); however, in many cases, the available monitoring data are scarce and cannot identify a clear decreasing concentration tendency, inducing incorrect interpretations [58,59]. Further research on bacterial community structure research can provide important data for BTEX biodegradation. Therefore, biodegradation kinetics should be considered in long-term prediction models of groundwater table fluctuations.

## 5. Conclusions

This study provides important insights into the migration behavior of light non-aqueous phase liquids (LNAPLs) in porous media, aiding the design and implementation of more efficient in situ remediation strategies and optimizing the repair of BTEX compounds. Experiments on the degradation of organic pollutants under groundwater table fluctuations indicate the need for a better understanding of the differences in biodegradation kinetics between the groundwater table fluctuation zone and the saturation zone to effectively remediate benzene and toluene pollution in groundwater. The groundwater table simulation system investigates the hydraulic intervention on BTEX migration, as well as the control of representative subunits of the groundwater table fluctuation system according to different porous media characteristics, further enhancing the understanding of BTEX migration and the influence of geological media in shallow petroleum-contaminated groundwater. Overall, this study highlights the significant influence of GTF on benzene and toluene behavior in single- and double-lithology soil column environments. The main conclusions can be summarized as follows:

(1) As demonstrated, groundwater table fluctuations can promote considerable LNAPL dissolution and, consequently, significant changes in the BTEX concentration over time. The migration of benzene and toluene in a single-lithology soil column packed with sand is mainly affected by flushing due to the hydraulic force induced by water table fluctuations, causing dissolution and adsorption onto the porous media. However, benzene and toluene migration in a double-lithology soil column packed with sand and silt is significantly affected by retention due to the higher adsorption induced by the 10 cm silt. The effect of GTF is lower in the single-lithology soil column than in the double-lithology soil column.
(2) The migration of benzene and toluene in the GTF zone is complex, and there is no clear relationship between the contaminant concentration change trend and the water table fluctuation. However, the contaminant concentration near and in the saturated zone increases as the water table falls, and decreases as the water table rises, especially when the water table near the sample collection point and dissolution mainly correlate with the BTEX migration in the saturated zone.
(3) For a contaminated site with a single-lithology structure consisting of sand, GTF promotes downward BTEX migration, and the contaminant concentration in the saturated zone increases, which is why more attention should be paid to organic contaminant removal within the groundwater. A contaminated site with a double-lithology structure containing silt has more retention contamination with BTEX and is more suited to the removal of organic contaminants from the silt layer. The bacterial community structures in the two columns are very different, especially in the GTF zone, and further research can provide important data for BTEX biodegradation. Biodegradation kinetics should be considered in long-term prediction models of groundwater table fluctuations and should be better understood for the remediation of BTEX compounds.

## Figures and Tables

**Table 1:** The basic physical and chemical properties of media in experiment columns [40].

Porous Media Type	Sand	Silt
Media Size (mm)	0.5–1.25	0.075–0.1
Soil Bulk Density (g cm^−3^)	1.51	1.38
Initial Porosity	0.30	0.28
Initial Water Content (%)	20.5	24.8
Permeability Coefficient (cm s^−1^)	1.2 × 10^−3^	7.9 × 10^−4^
pH	8.51	8.45
Organic Matter Content (%)	0.78	3.33

**Table 2:** The physical and chemical properties of benzene and toluene [41].

Pollutant	Molecular Formula	Molecular Weight	Density (g cm^−3^, 20 °C)	Solubility in Water (mg L^−1^, 25 °C)	Log *K_ow_*	*K_oc_*
Benzene	C_6_H_6_	78.1	0.8765	1800	1.6~2.4	10~12.9
Toluene	C_6_H_5_CH_3_	92.1	0.8669	347~707	2.1~3.0	11.9~14.8

## Data Availability

The data that support the findings of this study are available from the corresponding author (Yang Y.) upon reasonable request.
